# Factors influencing patient preferences for obesity pharmacotherapy: The triangulation of semi-structured interviews, photovoice study and focus group discussions

**DOI:** 10.1016/j.obpill.2026.100256

**Published:** 2026-03-03

**Authors:** Alvin Mondoh, Francisca Contreras, Hilary Craig, Michael Crotty, Carel W. Le Roux

**Affiliations:** a Diabetes Complications Research Centre, Conway Institute of Biomedical and Biomolecular Research, University College Dublin, Dublin 4, Ireland; b My Best Weight Clinic, Dublin, Ireland

**Keywords:** Obesity, Pharmacotherapy, SOPHIA, Tirzepatide, Semaglutide, Patient preferences, Qualitative triangulation, Thematic analysis

## Abstract

**Introduction:**

Obesity is a complex and chronic disease associated with complications including type 2 diabetes, cardiovascular disease, and malignancy. Although pharmacotherapy has emerged as an effective treatment option, the determinants of patient preferences for specific pharmacological agents remain insufficiently characterized.

**Methods:**

This was a qualitative, multi-method, triangulated study designed to understand how adults living with obesity make decisions about initiating pharmacotherapy. Adults aged 18–70 years with a body mass index of ≥27 kg/m^2^ and at least one obesity complication were purposively recruited. All participants from semi-structured interviews (n = 15), Photovoice Study (n = 12) and focus group discussions (n = 12) were naive to obesity medications.

**Results:**

Tirzepatide was the most popular agent across all qualitative methods, primarily due to its clinically significant weight reduction. Semaglutide was the second most frequently selected option, attributed to its efficacy and widespread societal familiarity. Triangulation of data identified five principal factors shaping patient preferences for pharmacotherapy: a) Anticipated treatment efficacy, b) Adequacy of information and community supports, c) safety and tolerability profile, d) Treatment burden and lifestyle integration, e) Logistics and structural barriers. While perceived efficacy and information transparency were the most influential determinants, safety perceptions consistently moderated patient enthusiasm for treatment options.

**Conclusion:**

Patients demonstrated clear hierarchies in their preferences for pharmacotherapy, with tirzepatide and semaglutide receiving the strongest endorsement. These preferences were primarily influenced by perceived efficacy and the trustworthiness of medication information, while safety, practicality, and social context serve as supporting factors. Clinicians should engage in shared decision-making, collaborating with patients to develop treatment plans that align with individual information needs and social support systems.

## Graphical abstract

**Figure:**
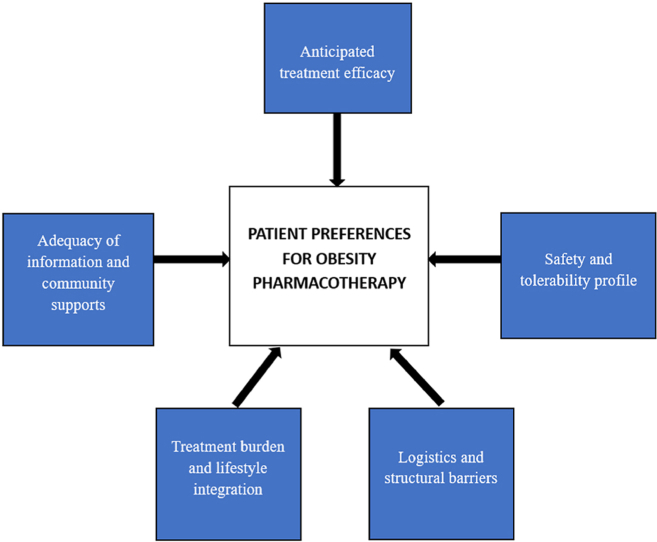

## 1 Introduction

Obesity is widely recognised as a complex and chronic disease characterised by biological, psychological, social, and environmental determinants rather than a simple imbalance between caloric intake and physical activity [1,2]. Over the past two decades, research has illuminated the neuroendocrine and metabolic mechanisms underlying appetite regulation and body-weight homeostasis [3,4]. These mechanisms strongly defend against weight reduction through hormonal adaptations that increase hunger, reduce satiety, and decrease energy expenditure following weight loss. In this context, obesity manifests as a biologically defended state, shaped by powerful physiological processes and complex interactions with obesogenic environments [5,6]. This evolving understanding has shifted global and clinical perspectives, reframing obesity as a chronic disease requiring long-term, multimodal management.

The global burden of obesity reflects this complexity. More than one billion individuals worldwide currently live with obesity, a figure expected to rise to nearly two billion by 2035 [7,8]. No country is on track to meet World Health Organization targets for obesity reduction. Europe faces one of the highest regional burdens, with approximately 59% of adults having overweight or obesity and nearly one-quarter meeting criteria for obesity [9]. Ireland reflects similar trends, with 60% of adults living with excess body weight and 25% meeting criteria for obesity [10]. Socioeconomic disparities continue to widen, with obesity disproportionately affecting individuals in lower-income households. These patterns show the inadequacy of behavioural approaches alone and highlight the need for accessible, biologically informed treatments aligned with patient needs and preferences.

Obesity has profound multisystem implications. Cardiometabolic complications remain the most prominent, including hypertension, dyslipidaemia, insulin resistance, type 2 diabetes, myocardial infarction, and stroke [11]. Visceral adiposity contributes directly to systemic inflammation through increased secretion of interleukin-6 (IL-6), tumour necrosis factor-alpha (TNF-α), leptin, and resistin, alongside reduced adiponectin, promoting endothelial dysfunction and accelerated atherosclerosis [12,13]. Obesity also contributes to a distinct cardiac phenotype known as obesity-related cardiomyopathy and has emerged as a leading contributor to heart failure with preserved ejection fraction (HFpEF), a metabolic–inflammatory syndrome characterised by impaired diastolic relaxation, microvascular inflammation, pulmonary hypertension, and reduced exercise capacity [14].

Respiratory complications include obstructive sleep apnoea (OSA), obesity hypoventilation syndrome, and reduced lung volumes due to mechanical restriction of the chest wall. These conditions contribute to arrhythmias, impaired functional capacity, and increased cardiovascular risk [15]. Hepatic complications such as metabolic dysfunction-associated steatotic liver disease (MASLD) and steatohepatitis (MASH) now constitute leading indications for liver transplantation globally [16]. Renal complications driven by glomerular hyperfiltration and ectopic fat deposition further exacerbate morbidity. Musculoskeletal issues including osteoarthritis, chronic pain syndromes, and limited mobility compound functional decline and perpetuate weight gain [17]. Reproductive complications include menstrual irregularities, infertility, gestational diabetes, adverse pregnancy outcomes, and male hypogonadism [18,19]. Obesity is also causally associated with at least 13 cancers, including endometrial, colorectal, renal, and postmenopausal breast cancers [20]. Finally, psychological and social harms such as depression, anxiety, disordered eating, body image distress, and internalised weight stigma significantly shape disease experience and treatment engagement [21,22].

Given the chronic, biologically defended nature of obesity, multimodal treatment approaches are required. Lifestyle interventions, though foundational, typically yield modest and difficult-to-maintain weight loss due to compensatory neuroendocrine adaptations that persist for years [23]. Bariatric surgery remains the most effective intervention for severe obesity, producing 25–35% long-term weight loss and metabolic improvements, but it is not acceptable or appropriate for all individuals and is limited by access and surgical risk [24]. Pharmacotherapy therefore plays a critical role in bridging the treatment gap for individuals requiring meaningful weight loss who cannot achieve or sustain reduction through behavioural strategies alone.

Early obesity medications such as orlistat, a reversible gastrointestinal lipase inhibitor, reduce dietary fat absorption by approximately 30%, resulting in 3–5% weight loss but are limited by gastrointestinal side effects, affecting adherence and acceptability [25]. Naltrexone/bupropion acts centrally on pro-opiomelanocortin (POMC) neurons, inhibiting reward-driven eating and enhancing satiety, producing 5–9% weight loss in phase III trials [26]. The emergence of incretin-based therapies marked a major advance. Liraglutide 3.0 mg, a daily GLP-1 receptor agonist, produces 8–10% weight loss and improves glycaemic control and cardiometabolic risk factors [27]. Semaglutide 2.4 mg, a weekly GLP-1 receptor agonist, produces approximately 15% weight loss across STEP trials, with additional reductions in blood pressure, inflammation, and hepatic steatosis [28]. The SELECT trial demonstrated a 20% reduction in major cardiovascular events, marking the first pharmacotherapy for obesity to show cardiovascular benefit independent of weight loss itself [29].

Tirzepatide, a dual GIP/GLP-1 receptor agonist, represents a paradigm shift. SURMOUNT-1 demonstrated 15–21% weight loss depending on dose [30]. GIP receptor activation may enhance metabolic flexibility, fat oxidation, and energy balance, produce synergistic effects when combined with GLP-1. Individuals rapidly regain weight after discontinuation, further illustrating the chronic nature of obesity. Emerging agents including oral semaglutide at obesity-specific doses; CagriSema (cagrilintide + semaglutide); survodutide, a GLP-1/glucagon dual agonist; and retatrutide, a GLP-1/GIP/glucagon triple agonist has demonstrated unprecedented efficacy, with retatrutide achieving nearly 24% weight loss in phase II data [31]. At the precision-medicine end of the spectrum, setmelanotide targets MC4R agonist, targets specific defects in the melanocortin pathway. It produces substantial weight loss in monogenic obesity due to pro-opiomelanocortin (POMC) deficiency and leptin receptor (LEPR) deficiency, where mean body mass index (BMI reductions of −26% have been observed in clinical trials, with a 45–80% rate of achieving ≥10% weight loss (Table 1) [32].

Understanding patient preferences has thus become essential to tailoring obesity care. Evidence from chronic disease management shows that alignment between patient priorities and treatment characteristics improves adherence, satisfaction, and long-term outcomes [33]. In obesity, preferences are shaped by expectations of weight loss, dosing convenience, perceived safety, cost, side effect profiles, injection burden, previous experiences with weight loss, and personal beliefs about obesity treatment. Qualitative evidence reveals that emotional, contextual, and identity-related factors such as stigma, hope, fear, mistrust, and previous healthcare interactions strongly shape treatment decisions [34]. Quantitative discrete-choice experiments demonstrate that individuals tend to prioritise efficacy and side-effect burden but cannot capture the deeper experiential narratives underpinning these choices [35,36].

The SOPHIA programme, funded under the Innovative Medicines Initiative, has advanced understanding of lived experiences of obesity and treatment preferences. Hilary Craig's work within SOPHIA identified the importance of psychological safety, trust, stigma, and emotional responses to treatment pathways, emphasising the need for personalized and compassionate obesity care [37]. However, these studies explored treatment preferences across modalities rather than between specific obesity medications.

Given the rapid expansion of pharmacotherapy and the gaps in current evidence, the present study aimed to explore how individuals living with obesity interpret and evaluate specific obesity medications. A triangulated qualitative approach that informed a three-part doctoral research series exploring patient preferences for obesity treatment through complementary qualitative methods was selected to capture the emotional, cognitive, contextual, and social influences guiding treatment choice. The three phases included semi-structured interviews, Photovoice Study and focus group discussions. Using these three different methodologies enabled data triangulation, provided unifying themes and a layered understanding of how patients make treatment decisions. In the first phase, qualitative analysis of semi-structured interviews identified five major themes (a) Effectiveness of medication, (b) Information to make decision (c) Safety of medications, (d) Practicality and (e) Individual strategies and Community supports in obesity management [38]. The four identified themes in the second phase of the Photovoice Study included a) Logistics around Lifestyle, b) Effectiveness, c) Safety, risk and tolerability and d) Accessibility [39]. In the third phase of focus group discussions the themes included (a) Holistic effectiveness beyond weight loss, (b) Treatment integration and regimen burden, (c) Safety, tolerability and social risk, (d) Structural and economic accessibility and (e) Navigating the information landscape.

## 2 Methods

### 2.1 Study design

This qualitative study used triangulation to provide a comprehensive understanding of patient preferences for obesity pharmacotherapy. Triangulation integrates multiple qualitative data sources to enhance credibility, identify convergent and divergent findings, and capture dimensions of experience that may not emerge from a single method [40,41]. The study combined semi-structured interviews, photovoice and focus group discussions to explore how individuals conceptualise obesity medications and what factors influence their choices.

A qualitative research design using reflective thematic analysis to explore patient preferences for obesity medication was also used. This enabled the capture of the depth and complexity of individuals' experiences and preferences, particularly in relation to their personal health and decision-making processes. Thematic analysis was conducted both inductively (allowed themes to emerge from the data itself) and deductively (guided by pre-existing theoretical frameworks, such as the Health Belief Model [42].

### 2.2 Participant recruitment

Purposive sampling was used to recruit the participants of the study (semi-structured interviews (n = 15), Photovoice Study (n = 12) and Focus group discussions (n = 12). Participants across all qualitative phases were adults aged 18–70 years and up to 75 years in the Photovoice Study) with a body mass index (BMI) ≥27 kg/m^2^ and at least one weight-related complication, who were treatment-naive to obesity pharmacotherapy but expressed interest in initiating medication. Participants were recruited from a specialist obesity clinic in Dublin, Ireland. The samples were predominantly female and also a few men were included across the phases. Commonly reported weight complications included hypertension, pre-diabetes, dyslipidaemia, type 2 diabetes, sleep apnoea, gastroesophageal reflux disease, depression, gallbladder disease and infertility (Table 2). Ethnicity data were not collected due to relative homogeneity of the study population and to maintain participant confidentiality given the small sample sizes. While the participant samples were independent across the three phases, identical inclusion criteria were applied to enable methodological triangulation.

Exclusion criteria included pregnancy, breastfeeding, severe psychiatric illness, and previous or planned bariatric surgery. Participation in the study was entirely voluntary, and no incentives or payments were provided to participants for their time. Ethical approval was obtained from University College Dublin (LS-23-74-LeRoux) and participants were provided with a written informed consent.

### 2.3 Data collection

To ensure a consistent baseline understanding of obesity medications, participants viewed a standardised 10-min educational video outlining approved pharmacotherapies in Europe namely orlistat, naltrexone/bupropion, liraglutide, semaglutide 2.4 mg, and tirzepatide as well as emerging agents including oral semaglutide, CagriSema, survodutide, orlistat, naltrexone/bupropion, liraglutide and retatrutide. The video described mechanisms of action, dosing schedules, trial-based efficacy, and side-effect profiles. This standardisation minimised informational bias and ensured that participants could make meaningful comparisons.

Semi-structured interviews served as the first data collection method. These were conducted via Zoom, each lasting approximately 45–60 min. Interviews explored participants’ understandings of obesity, previous experiences with weight loss, expectations for pharmacotherapy, emotional reactions to specific medications, perceived barriers, safety concerns, and contextual influences [43].

Photovoice is a participatory method that empowers individuals to document lived experiences through photography, providing insights into contexts, beliefs, emotions, and environmental influences that shape decision-making [44]. Participants were invited to take photographs over a two-week period that symbolized their reasons for preferring particular obesity medications. They were instructed to avoid photographing identifiable individuals and instead capture symbolic, emotional, or contextual representations. Cameras were returned in prepaid envelopes and photographs were printed for use during interviews. Photographs taken during the Photovoice Study were used as elicitation tools. This approach aligns with methodological guidance enabling deeper reflection and richer narrative data [45].

Two in-person focus group discussions were conducted, each consisting of six participants. Focus groups allowed exploration of shared experiences, social comparison, and peer-influenced perspectives, revealing insights that may not emerge in individual interviews [46,47]. Participants viewed the same educational video at the beginning of each focus group discussion to maintain consistency in knowledge base.

### 2.4 Data analysis

All sessions were audio-recorded, anonymised, and transcribed verbatim. Data were analysed using reflexive thematic analysis as outlined by Braun and Clarke [48,49]. Two researchers independently coded transcripts using MAXQDA 2024, employing both inductive and deductive approaches guided by the Health Belief Model and findings from the SOPHIA qualitative studies. Coding discrepancies were resolved through iterative discussion.

Triangulation was achieved by integrating insights across the three methods. Convergence highlighted themes appearing consistently across semi-structured interviews, Photovoice Study and focus groups discussions. Complementarity referred to themes that enriched each other across methods. For instance, the semi-structured interviews were contextualized, the Photovoice Study illuminated emotional or symbolic dimensions while the focus groups revealed social dynamics influencing medication preferences. Divergence captured tensions or conflicting viewpoints, offering deeper understanding of variability and reinforcing analytical rigour.

Ethical considerations included ensuring confidentiality, managing potentially distressing discussions about obesity and stigma, and following trauma, informed interviewing principles. Photographs were handled with care to ensure privacy, and participants were reminded that they could withdraw at any time.

This triangulated qualitative design enabled a multidimensional exploration of how adults living with obesity evaluate and choose between medications in an era of rapidly evolving pharmacotherapy. The integration of visual, narrative, and group-based methods provides a robust foundation for understanding patient preferences and informing clinical decision making.

## 3 Results

**Fig. 1:**
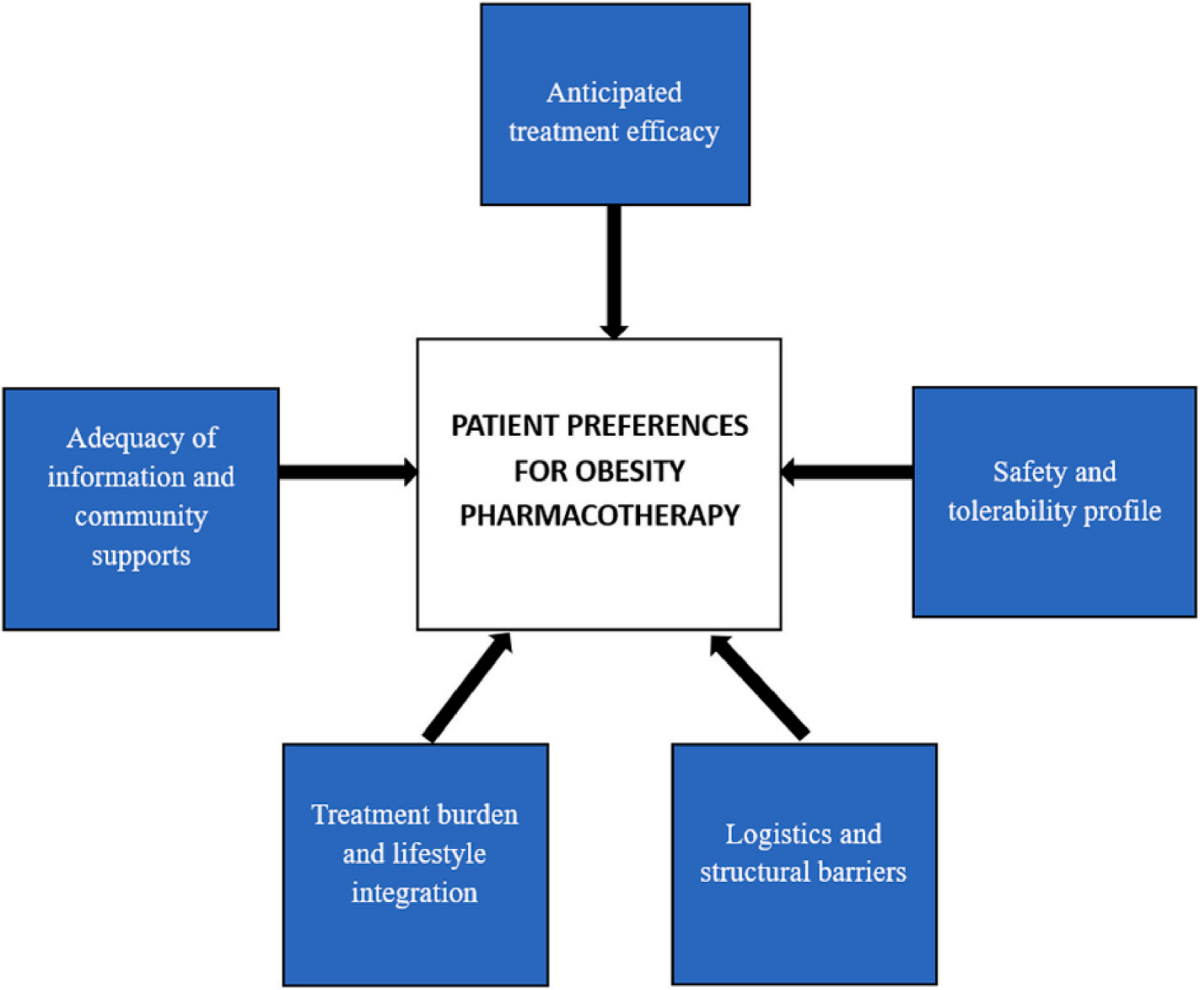
Overview of triangulated themes.

The triangulation of data identified five principal factors shaping patient preferences for pharmacotherapy: a) Anticipated treatment efficacy, b) Adequacy of information and community supports, c) Safety and tolerability profile, d) Treatment burden and lifestyle integration, e) Logistics and structural barriers as in Figure 1.

### 3.1 Patient characteristics

Patient characteristics across the three qualitative studies are summarised in Table 2.

### 3.2 Synthesis of findings across the three methodologies

**Fig. 2:**
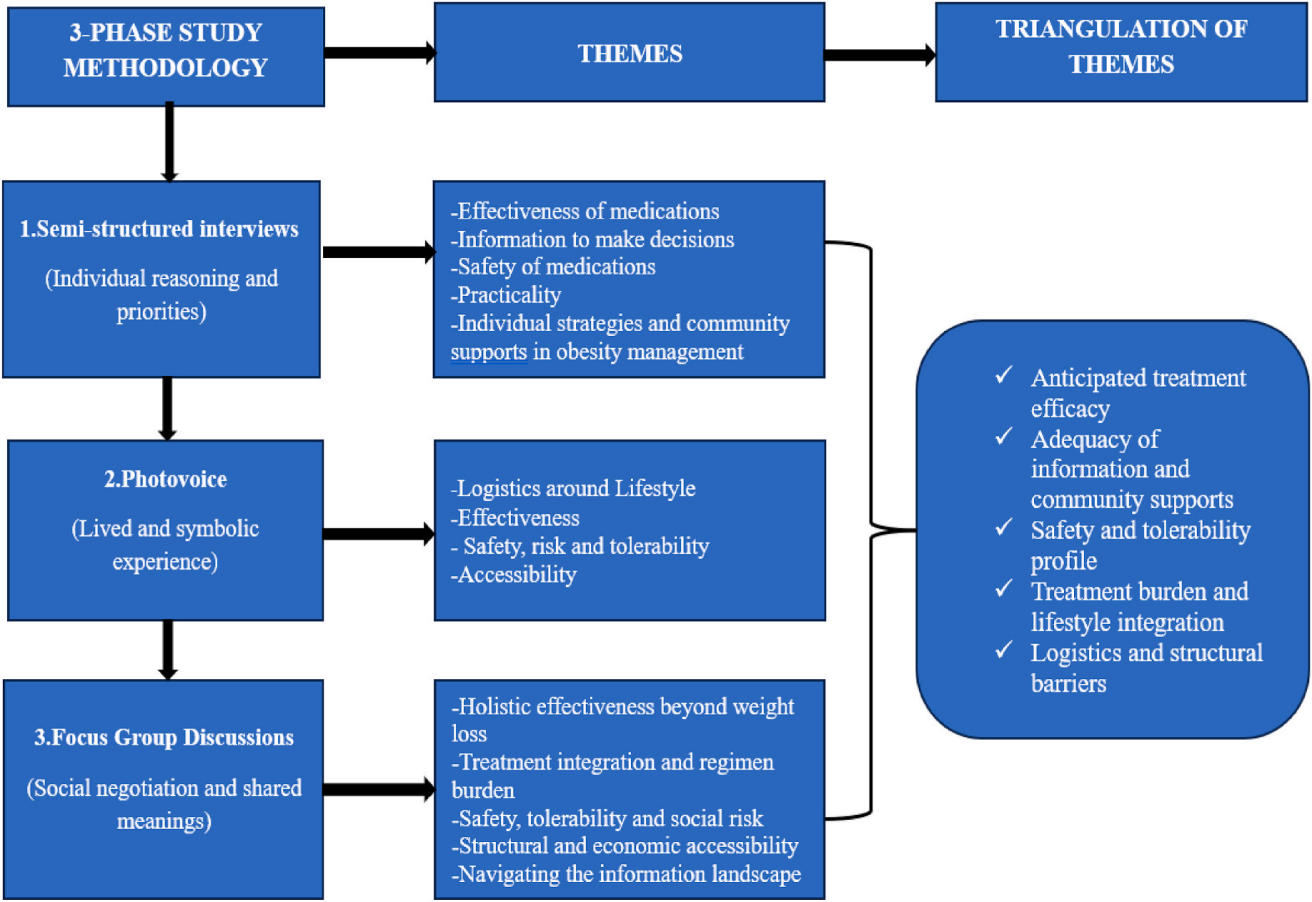
Synthesis of findings across the three methodologies.

The three studies revealed consistent, overlapping themes while also providing unique insights afforded by each methodological approach (Fig. 2). The semi-structured interviews provided deep personal narratives, identifying five core themes:(a) Effectiveness of medications, (b) Information to make decisions, (c) Safety of medications, (d) Practicality and (e) Individual strategies and community supports in obesity management. Patients emphasized the importance of weight loss and health improvement but equally valued psychological relief from “food noise” and the emotional burden of obesity. Safety concerns particularly regarding gastrointestinal side effects, emerged as a major barrier to treatment initiation and adherence.

The Photovoice Study visually enriched themes, revealing how preferences are embedded in daily life. The four themes identified included a) Logistics around Lifestyle, b) Effectiveness, c) Safety, risk and tolerability and d) Accessibility and they were illustrated through powerful imagery like medications and clothing. This method highlighted the material and symbolic realities of living with obesity, such as the desire for discreet treatment, fear of social stigma and the financial burden of long-term therapy.

Focus Group discussions fostered interactive dialogue, revealing how social dynamics and shared experiences shaped perceptions. Five themes emerged; (a) Holistic effectiveness beyond weight loss, (b) Treatment integration and regimen burden, (c) Safety, tolerability and social risk, (d) Structural and economic accessibility and (e) Navigating the information landscape. Participants compared experiences, debated preferences and collectively critiqued healthcare communication and societal stigma. The participants indicated the importance of peer validation and the shared struggle against misinformation and judgement.

Together these studies presented a coherent picture of patient decision-making in obesity pharmacotherapy which is a multidimensional, dynamic process shaped by the interplay of clinical efficacy, personal and logistical feasibility, perceived safety, social context and systemic barriers.

### 3.3 Overview of triangulated themes

Triangulation across the semi-structured interviews, photovoice and FGDs demonstrated a strong convergence across core themes relating to anticipated treatment efficacy, safety and side effects concerns, adequacy of information, stigma, autonomy in decision-making and the role of health care professionals. Method specific themes were also identified including visual embodiment of weigh and identity within the Photovoice Study and collective sense-making through group interaction within focus group discussions highlighting the complementary contribution of each qualitative approach (Table 3).

Triangulation of the data identified five principal factors shaping patient preferences for pharmacotherapy: a) Anticipated treatment efficacy, b) Adequacy of information and community supports, c) safety and tolerability profile, d) Treatment burden and lifestyle integration, e) Logistics and structural barriers (Figure 2).

To enhance clinical translation of our findings, the five principal factors influencing patient preferences are summarised in Table 4. This framework synthesizes patient concerns identified across semi-structured interviews, Photovoice Study and focus group discussions, alongside practical communication strategies for shared decision-making. By mapping patient priorities, the table provides a concise tool for clinicians to explore what matters to most individuals seeking obesity pharmacotherapy. Incorporating these domains into routine consultations may improve treatment acceptance, adherence and long-term outcomes.

#### 3.3.1 Anticipated treatment efficacy

This theme revealed that patients defined treatment success in broad personally meaningful terms that extend far beyond numerical weight reduction. In the semi-structured interviews, effectiveness was discussed in relation to specific weight loss targets often between 15% and 25%, impact on obesity related complications and mental health improvements with particular emphasis on reducing “food noise”. The Photovoice Study added tangible, symbolic depth to this concept with photographs of calculators, cherished clothing and family-related items which tied effectiveness to deeply personal goals such as fitting into old clothes, achieving pregnancy, reducing cardiovascular risk and enhancing physical mobility. The FGDs further reinforced that cognitive freedom from constant thoughts about food was frequently prioritized as much as or even more than weight loss itself, with group dialogue underscoring the variability in how individuals define a successful outcome.

Moreover, across the three qualitative methods, tirzepatide emerged as the most preferred pharmacotherapy agent, selected by approximately 50% of participants in the Photovoice Study. Participants cited its superior weight loss efficacy (15%–21%) in trials, perceived synergy due to dual GIP/GLP-1 receptor agonism and its alignment with personal health goals such as reducing cardiovascular risk and improving reproductive health. Semaglutide was the second most favoured option preferred for its established efficacy of 15% weight loss, widespread familiarity and perceived safety due to longer clinical exposure. In contrast, orlistat and naltrexone bupropion received limited endorsement due to concerns over gastrointestinal side effects, modest weight loss (3%–9%) and tolerability issues such as insomnia and diarrhea. Emerging agents still in phase three trials like CagriSema, retatrutide, survodutide, generated interest but were less preferred due to limited familiarity and concerns about long-term safety data. The preferences were visually and narratively enriched through photovoice, where tirzepatide was symbolized through images of calculators that targeted weight loss, pacifiers for reproductive goals and family memorabilia for longevity concerns. Semaglutide was associated with appetite control, depicted through food imagery such as pancakes, representing the desire to reduce “food noise”. Orlistat and naltrexone/bupropion were linked to photographs of toilets and medication boxes, reflection fears of digestive distress and polypharmacy.

The FGDs further contextualized these preferences by revealing how social dynamics, shared experiences and peer comparisons shaped perceptions of specific agents. Participants debated the merits of tirzepatide versus semaglutide not only in terms of weight loss percentages but also in relation to real world factors such as injection frequency, social stigma and media portrayal. The collective critique of societal stigma also moderated enthusiasm for GLP-1 based therapies with some participants hesitant to choose medications widely discussed in media as “easy fixes”. FGDs also highlighted how anecdotal evidence from peers such as personal stories of side effects or success with naltrexone/bupropion directly influenced willingness to try or avoid certain medication preferences are socially negotiated and not merely individually calculated.

This triangulated view confirmed that effectiveness is a personalized construct that integrates biometric, emotional and social dimensions where psychological relief is often equally valued as physical change.

#### 3.3.2 Adequacy of information and community supports

This highlighted the central role of knowledge, communication and health literacy in patient decision making, In the interviews, participants stressed the importance of receiving clear, comprehensive information from healthcare providers, particularly general practitioners to make informed choices while also acknowledging the influence of social media and personal networks. The Photovoice Study, though less explicitly focused on information sourced, implicitly revealed participants’ engagement with medication information through images of drug leaflets and instructions, reflecting a process of personal research and verification. The FGDs provided the richest insights into this theme, vividly illustrating the tension between seeking credible professional guidance and relying on accessible but often unreliable digital and social sources. Participants described a proactive yet cautious approach to information seeking, valuing transparency from clinicians but also cross-referencing advice with tangible outcomes observed within their personal networks. This triangulation revealed that patients are active, critical consumers of health information who operate within a complex ecosystem of formal and informal sources, and who trust is ultimately built on a combination of professional expertise, personal evidence and relatable peer experiences.

#### 3.3.3 Safety and tolerability profile

This emerged as a major barrier to both initiating and continuing medication with concerns articulated across clinical, personal and social dimensions. In the semi-structured interviews, participants frequently cited side effects such as nausea, insomnia and gastrointestinal disturbances and expressed a preference for medications with longer established safety records. The Photovoice Study powerfully conveyed the embodied experience of risk through visual metaphors with images of gallstones, toilets and personal medication boxes made the personal and physical reality of side effects vividly apparent. FGDs added a critical social layer to this theme as participants reported feeling stigmatized and judged for using obesity pharmacotherapy, a sentiment that compounded their perceptions of risk and influenced their treatment choices. This convergence of findings emphasized that safety is not solely a clinical issue but a psychosocial experience, where risk perception is shaped by an interplay of medical evidence, personally bodily experience and societal narratives.

#### 3.3.4 Treatment burden and lifestyle integration

This theme emerged across all methods reflecting how patients evaluate medications based on compatibility with their daily routines, work schedules and personal lives. In the interviews, participants emphasized practical considerations such as preferences for weekly over daily dosing and oral over injectables, though the hypothetical scenario of cost free access shifted some focus towards efficacy and safety. The photovoice component enriched this understanding by visualizing the lived reality of medication integration of images of workstations and oral medications that highlighted the importance of discreet and manageable regimens with weekly injections often symbolized as less intrusive to daily life. During the FGDs, participants engaged in social and practical negotiations around dosing frequency with some expressing concern about forgetting daily doses while others valued the perceived steady control offered by daily administration. Together, these perspectives illustrated that lifestyle fit is not merely a logistical concern but a primary determinant of both initial acceptance and long-term adherence to obesity medication.

#### 3.3.5 Logistics and structural barriers

This theme persisted throughout participants narratives, reflecting deeply ingrained concerns about equity and real-world access of medication. During interviews, affordability and availability were acknowledged as secondary, yet participants still emphasized the importance of reliable supply and systemic access. Photovoice gave a symbolic form to these concerns with photographs of Euro banknotes, drug payment scheme cards and a teddy bear with one eye closed which visually articulated anxieties about long term cost, supply insecurity and ethical dilemmas during medication shortages. In FGDs, discussions extended to systemic gaps such as lack of awareness, socioeconomic barriers and the gatekeeping role of healthcare providers. This multilayered understanding highlighted that accessibility encompassed financial, logistical and informational dimensions and that even if cost is removed, emotional and ethical barriers related to access remained significant and will eventually influence a patient's decision making.

The triangulation of these three qualitative approaches revealed that patient decision making in obesity pharmacotherapy is a dynamic and context dependent process. The semi-structured interviews provided depth into individual reasoning and priorities, the Photovoice Study uncovered the embodied and symbolic dimensions of choice and the FGDs illuminated the social and interactive construction of preferences. Collectively, they offered a comprehensive framework for understanding how patients navigate the complex interplay of treatment efficacy, personal lifestyle, perceived risk and structural reality. These integrated findings stressed on the necessity of a patient-centred, shared decision-making model in obesity care that addresses not only weight loss but also lifestyle integration safety and tolerability, psychological well-being and equitable access.

## 4 Discussion

This study provided a nuanced, multidimensional understanding of patient preferences for obesity pharmacotherapy by triangulating data from semi-structured interviews, photovoice and focus group discussions. The findings confirmed that patient decision making is a complex, context dependent process that extends far beyond a simple calculus of weight loss efficacy.

Our results reinforced and extended prior SOPHIA consortium research shifting the focus from general treatment modalities to the specific attributes of pharmacological agents [37]. The findings challenged the dominant biomedical paradigm that prioritizes percentage weight loss as the primary, if not sole, metric of success in obesity pharmacotherapy. While anticipated weight reduction was a powerful driver explaining the strong preference for tirzepatide and semaglutide, the triangulation revealed that participants defined effectiveness in profoundly holistic and personal terms. The clear preference hierarchy with tirzepatide and semaglutide as the most favoured was primarily driven by perceptions of superior efficacy and psychological relief from “food noise” aligning with clinical trial data [28,30]. This aligns with emerging literature on the significant impact of GLP-1 based therapies on cravings and obsessive food thoughts which are major contributors to obesity disease burden [38]. Our study therefore supports and extends calls for the systematic inclusion of Patient Reported Outcome Measures (PROs) and the quality of life assessments in obesity clinical trials and clinical practice guidelines [50]. A “Whole Life Efficacy” framework, as proposed, would better capture the therapeutic goals that atter most to patients, moving from a model of weigh management to one of health and life re-engagement.

While the interviews provided depth on personal risk-benefit calculations, the FGDs revealed that preferences are fluid and shaped by peer comparison and collective sense making. Participants actively debated the merits of medications weighing anecdotal evidence from social networks against official information. The shared critique of societal stigma particularly the perception of pharmacotherapy as an “easy way out” moderated the enthusiasm for some highly effective agents. This finding highlighted that medication choice occurs within a social ecology meaning where trust is built not only on clinical data but also on relatable peer experiences and the perceive legitimacy of the treatment within one's social circle [35,51].

The theme of safety and tolerability was amplified through social discourse. Concerns about gastrointestinal side effects were not just worries but were imbued with fear of social stigma and judgement, a dimension vividly captured in the photovoice images. This triangulation of data confirmed that risk perception is psychosocial, shaped by an interplay of bodily experience, medical information and societal narrative [22,52]. Healthcare providers must therefore be prepared to address not only the physiological risks of side effects but also the associated social and emotional risks that can deter treatment initiation and adherence.

Across all the three methods but most starkly in the FGDs, participants articulated how their choices were constrained by logistical and economic burden. Anxiety about long term affordability, frustration with supply chain instability and experiences with healthcare providers lacking contemporary knowledge were not peripheral concerns but central factors in decision making [53,54]. This revealed a fundamental tension of which patients are encouraged to be active agents in shared decision making yet they operate within systems that significantly limit their options. These findings demonstrated the need for a parallel policy-level action to ensure equitable access, stable supply and adequate healthcare provider education.

### 4.1 Implications for clinical practice

The findings of this research have direct implications for healthcare providers, educators and healthcare systems delivering obesity care. It is essential to adopt a Shared Decision-Making (SDM) Model tailored specifically to obesity pharmacotherapy, where clinicians move beyond prescribing based solely on clinical indicators [55,56]. SDM conversations should explicitly explore patient defined goals by asking, “What does success look like for you beyond weight loss?“; consider lifestyle integration through questions such as, “How would a weekly injection fit into your routine compared to a daily tablet?“; establish tolerability thresholds by inquiring, “What side effects would be most disruptive to your daily life?“; and address cost and access realities with, “What are your concerns about affordability or ongoing supply?“.

Healthcare providers must provide balanced, transparent and accessible information as patients seek clarity on benefits, risks and variability in response. This involves offering standardized, evidence-based information similar to the video used in these studies to ensure a baseline understanding, acknowledging uncertainties including individual variation in response and long-term data gaps and proactively discussing strategies for managing common side effects to reduce anxiety and prevent early discontinuation. Furthermore, healthcare providers play a key role in countering societal stigma by using non-stigmatising language, validating obesity as a chronic disease requiring long term management and creating a clinical environment where patients feel heard and not judged for seeking pharmacological help. Finally developing integrated multidisciplinary care pathways is crucial as effective obesity management requires more than a prescription. Care models should facilitate access to dietitians, psychologists, physiotherapists and support groups as emphasized by participants who value community and holistic support.

### 4.2 Implications for health policy

The structural barriers identified necessitates comprehensive policy-level action to ensure effective and equitable obesity management. Policymakers must develop sustainable reimbursement models to improve affordability and address the risk of a two-tier system created by high medication costs. This requires health economic analyses that capture broader societal benefits such as reduced complications and improved productivity to justify investments. Simultaneously, policy interventions are needed to ensure stable supply chains, as shortages driven by demand and manufacturing issues undermine treatment continuity and create ethical dilemmas. Additionally, national and health programmes must mandate and fund ongoing education for general health practitioners and frontline healthcare providers to close primary care knowledge gaps regarding contemporary treatments including pharmacotherapy. Finally, public health literacy campaigns should be supported to combat misinformation and stigma by promoting an accurate understanding of obesity as a complex chronic disease and the legitimate role of medication in its management.

This research makes several important contributions to the literature on obesity management and patient-centred care. First, it extends previous SOPHIA research by moving beyond general treatment preferences to focus specifically on pharmacotherapy decision making. While earlier studies highlighted the importance of efficacy, safety and support, this programme details how patients weigh attributes like dosing frequency, route administration, side effect profiles and cost when choosing between specific medications [34,38]. This granularity is critical as the therapeutic landscape expands with new agents such GLP-1 and dual GIP/GLP-1 receptor agonists.

Second, the findings challenge a purely biomedical model of obesity treatment that prioritizes weight loss metrics above all else. Participants consistently defined effectiveness holistically valuing reduced psychological food control, improved mental well-being, enhanced mobility and social participation as much as or sometimes more than the percentage of weight loss. This aligns with growing calls for patient reported outcomes and quality of life measures in obesity trials [50]. Our work suggests that “success” should be reframed using what might better be termed as a “Whole-life Efficacy” framework which integrates biomedical, psychological and social dimensions of health.

Third, the research highlighted the underappreciated role of structural and systemic barriers in shaping “preferences”. What may appear as a patient's choice is often a constrained decision made within contexts of limited access, high cost, supply instability and inadequate clinical guidance. Participants expressed anxiety about affordability, ethical concerns about medications shortages and frustration with poorly informed healthcare providers. These findings resonate with health policy literature on inequities in obesity care [57] and highlights that patient preference cannot be realised without addressing structural determinants of access.

Finally, the use of the photovoice methodology in obesity pharmacotherapy research is novel and generative. By enabling participants to create and reflect on images, the study captured embodied, emotional and symbolic dimensions of medication choices that are difficult to convey in words alone. This approach aligns with broader moves towards participatory health research and offers a model for engaging patients as co-producers of knowledge.

### 4.3 Strengths and limitations

Strengths of this research programme included methodological triangulation where the use of three qualitative methods provided comprehensive, convergent and richer insights than any single approach could achieve. The triangulation of these methods strengthened the credibility and richness of the findings by allowing themes to be validated across different forms of data. This was supported by a participatory and patient-centred design which particularly through the photovoice component empowered participants as active contributors rather than passive subjects. Discrepancies between phases such as the more pronounced emphasis on cost in FGDs compared to interviews were analytically productive, revealing how research context and method can shape participant expression. The rigour of the analysis was further ensured through independent coding, team discussion and the application of established theoretical frameworks, enhancing the overall trustworthiness of the findings. The consistent use of MAXQDA 24 software across all phases supported rigorous cross-comparison and theme refinement ensuring a transparent, reproducible and thoroughly documented analytical process.

However, several limitations must also be acknowledged. The sample characteristics being predominantly female and Irish, treatment naive and recruited from a single specialist centre limited the generalizability of the findings to other cultural, socioeconomic or clinical contexts. Furthermore, the cross-sectional design captured preferences at only a single and pre-treatment point in time.

Therefore, future research should adopt a longitudinal approach to track patients from pre-treatment through initiation and long-term maintenance, understanding how experiences and adherence evolve. It should also expand to include comparative studies exploring how different healthcare systems and cultural attitudes shape medication preferences alongside intervention development to design clinical tools like decision aids based on the identified themes. Finally, details of health economics research are required to conduct cost-effectiveness analyses of newer medications within public healthcare systems to inform sustainable funding models.

## 5 Conclusion

Patients demonstrated clear hierarchies in their preferences for pharmacotherapy with tirzepatide and semaglutide receiving the strongest endorsement across interviews, photovoice and the focus group discussions. These preferences were primarily influenced by perceived efficacy and trustworthiness of medication information while safety, practicality and social context served as supporting factors. Orlistat and naltrexone/bupropion were less favoured due to tolerability concerns and modest weigh loss. Emerging agents, while of interest were approached with caution due to limited familiarity. These findings highlight the importance of transparent, evidence-based communication and the inclusion of patient perspectives to enhance pharmacotherapy uptake and adherence.

### 5.1 Key takeaway messages

• Patient preferences for obesity medications are deeply personal, contextually embedded decisions that balance clinical hopes against logistical realities, weight benefits, social and financial costs.
• There should be a shift in obesity care from a healthcare-centred model focused on prescribing to a collaborative, patient-centred model focused on partnering.
• Healthcare providers and health systems can better support patients in navigating their treatment pathways by engaging in shared decision making, collaborating to develop treatment plans that align with individual information needs and support social systems ultimately improving adherence, outcomes and quality of life for people living with obesity.

## Ethics statement

This human study was approved by Human Research Ethics Committee – Sciences (UCD School of Medicine) with approval number LS-23-74-LeRoux. All adult participants provided written informed consent to participate in this study. Written informed consent was obtained from the individual(s) for publication of the details of their medical case.

## Author contributions

(AM) Alvin Mondoh: Conceptualization, Methodology, Investigation, Data Analysis, Writing - Original Draft, Project Administration.

(FC) Francisca Contreras: Data Curation, Writing - Review & Editing.

(HC) Hilary Craig: Writing - Review & Editing.

(MC) Michael Crotty: Supervision, Data Analysis, Writing - Review & Editing.

(CW) Carel W. le Roux: Supervision, Conceptualization, Funding acquisition, Writing - Review & Editing.

## Declaration of AI use

The authors declare that they have not used any type of generative artificial intelligence for writing of this manuscript, nor for the creation of images, graphics, tables or their corresponding captions.

## Funding sources

SOPHIA has received funding from the Innovative Medicines Initiative 2 Joint Undertaking under grant agreement No. 875534. The funding source had no role in the study design, execution, data analysis, manuscript conception, planning, writing, or the decision to publish. All the authors of this paper are part of SOPHIA.

## Conflict of interest statement

The authors have no conflicts of interest to declare.

## Figures and Tables

**Table 1:** Summary of Current and Emerging Obesity Pharmacotherapies

Medication	Mechanism of Action	Mean Weight Loss	Side Effects	Regulatory Status
Orlistat	Gastrointestinal (GI) lipase inhibition	3–5%	Steatorrhoea, oily stools, malabsorption	European Medicines Agency/Food and Drug Administration (EMA/FDA approved
Naltrexone/Bupropion	POMC activation + reward modulation	5–9%	Nausea, insomnia, ↑BP	EMA/FDA approved
Liraglutide 3.0 mg	GLP-1 receptor agonist	8–10%	GI effects, gallbladder disease	EMA/FDA approved
Semaglutide 2.4 mg	GLP-1 receptor agonist	∼15%	GI effects, gallbladder	EMA/FDA approved
Tirzepatide	Dual GIP/GLP-1 RA	15–21%	GI effects, ↑HR	FDA approved
Oral Semaglutide (50 mg)	GLP-1 RA	∼15%	GI intolerance	Investigational
CagriSema	Amylin analogue + GLP-1 RA	15–17%	GI effects	Investigational
Survodutide	GLP-1/glucagon dual agonist	9–13%	GI effects, liver enzymes	Investigational
Retatrutide	Triple GIP/GLP-1/glucagon agonist	22–24%	GI effects, ↑Heart Rate (HR)	Investigational
Setmelanotide	MC4R agonist	25–50%	Hyperpigmentation, mood changes	EMA/FDA (rare obesity)

**Table 2:** Patient characteristics across qualitative study phases

Characteristic	Semi-structured interviews (n = 15)	Photovoice (n = 12)	Focus group discussions (n = 12)
**Age (Years)**	18–70	18–75	18–70
**Sex**	Female-10; Male-5	All female	Female-11; Male-1
**BMI**	≥27 kg/m^2^	≥27 kg/m^2^	≥27 kg/m^2^
**Treatment status**	Treatment-naïve to obesity medication	Treatment-naïve to obesity medication	Treatment-naïve to obesity medication
**Weight-related complications**	Hypertension, diabetes, dyslipidaemia, type 2 diabetes	Pre-diabetes, dyslipidaemia, hypertension, gallbladder disease, infertility, gastroesophageal reflux disease	Hypertension, prediabetes, dyslipidaemia, sleep apnoea, depression
**Clinical setting**	Specialist obesity clinic, Dublin, Ireland	Specialist obesity clinic, Dublin, Ireland	Specialist obesity clinic, Dublin, Ireland
**Ethnicity**	Not collected	Not collected	Not collected

**Table 3:** Triangulation of common themes across qualitative methodologies

Common themes	Semi-structured interviews	Photovoice	Focus group discussions
Anticipated treatment efficacy	✓	✓	✓
Adequacy of information and understanding treatment	✓	✓	✓
Safety and side effect concerns	✓	✓	✓
Stigma and moral judgment associated with obesity treatment	✓	✓	✓
Autonomy in decision-making	✓	✓	✓
Role of healthcare professionals in treatment decision-making	✓	✓	✓
Autonomy to initiate pharmacotherapy	✓	✓	✓
Practical and access related considerations (cost, accessibility and continuity)	✓	✓	✓
Visual embodiment of weight, identity and lived experience	x	✓	x
Collective sense-making and reassurance through group discussion	x	x	✓

**Table 4:** Factors Influencing Patient Preferences for Obesity Pharmacotherapy – Clinical Summary

Factor	Patient Concerns & Priorities	Clinical Implications & Communication Strategies
**Efficacy**	• Desire for 15–21% weight loss aligned with tirzepatide and semaglutide trial data • Psychological relief from “food noise” often valued as much as weight loss itself • Success defined holistically: improved mobility, reproductive goals, cardiovascular risk reduction, fitting into old clothes	• Present trial efficacy data transparently but frame within patient's personal goals • Ask: “What does success look like for you beyond the number on the scale?“. • Acknowledge that reduced food preoccupation is a valid and meaningful outcome.

**Information**	• Trust in healthcare professionals (especially GPs) balanced against pervasive social media influences • Active cross-referencing of professional advice with peer experiences and online sources • Desire for clear, balanced, and standardized education about benefits, risks, and response variability	• Provide standardized evidence-based information (e.g., brief educational videos or leaflets) • Acknowledge uncertainties openly (e.g., long-term data gaps, individual response variation) • Proactively discuss side effect management to reduce anxiety and prevent early discontinuation

**Safety & Tolerability**	• Fear of gastrointestinal side effects (nausea, diarrhea, gallstones) • Preference for agents with longer established safety records • Concerns compounded by fear of social stigma and judgment from others • Risk perception shaped by bodily experience, medical information, and societal narratives	• Address not only physiological risks but also associated social and emotional concerns • Normalize side effect discussions and provide practical management strategies • Validate that concerns about tolerability are legitimate and influence real-world adherence

**Lifestyle Fit**	• Preference for weekly over daily dosing; oral over injectable where possible • Need for discreet regimens that integrate seamlessly with work, travel, and social life • Concerns about forgetting daily doses versus perceived steady control from daily administration	• Explore regimen compatibility early: “How would a weekly injection fit into your routine compared to a daily tablet?” • Recognize that lifestyle integration is a primary determinant of both acceptance and adherence • Offer flexibility where options exist

**Access**	• Anxiety about long-term affordability and cost sustainability • Frustration with supply chain instability and medication shortages • Ethical dilemmas during shortages (who should receive limited supplies?) • Experiences with HCPs lacking contemporary obesity pharmacotherapy knowledge	• Acknowledge cost and access realities openly; discuss affordability concerns early • Advocate for patients by documenting need and supporting reimbursement applications • Stay updated on pharmacotherapy options through continuing education • Address emotional and ethical barriers related to access, not just financial ones

## Abbreviations

BMI –: Body mass index
GLP-1 –: Glucagon-like peptide-1
GIP/GLP-1RAs –: Glucose-dependent insulinotropic polypeptide/GLP-1 receptor agonists
SOPHIA –: Stratification of Obesity Phenotypes to Optimize Future Obesity Therapy
OSA –: Obstructive sleep apnoea
MASLD –: Metabolic dysfunction-associated steatotic liver disease
MASH –: Steatohepatitis
HFpEF –: Heart failure with preserved ejection fraction
POMC –: Pro-opiomelanocortin
MC4R –: Melanocortin-4 receptor
IMI –: Innovative Medicines Initiative
FGDs –: Focus Group Discussions
SDM –: Shared Decision-Making
PROs –: Patient reported outcome measures
LEPR: leptin receptor
GI –: Gastrointestinal
EMA –: European Medicines Agency
FDA –: Food and Drug Administration
HR –: Heart Rate
BP –: Blood pressure

## References

[1] Bruno F., Puleo C., Rizzo A. (2024). Obesity as a multifactorial disease: understanding and addressing stigma for holistic management. Adv Med Psychol Publ Health.

[2] Minari T.P., Manzano C.F., Yugar L.B.T., Sedenho-Prado L.G., de Azevedo Rubio T., Tácito L.H.B. (2025). Demystifying obesity: understanding, prevention, treatment, and stigmas. Nutr Rev.

[3] Yoo E.-S., Yu J., Sohn J.-W. (2021). Neuroendocrine control of appetite and metabolism. Exp Mol Med.

[4] Affinati A.H., Myers Jr MG. (2021).

[5] Bray G.A., Kim K.-K., Wilding J.P., Federation W.O. (2017). Obesity: a chronic relapsing progressive disease process. A position statement of the world obesity Federation. Obes Rev.

[6] Martínez-Gómez M.G., Roberts B.M. (2022). Metabolic adaptations to weight loss: a brief review. J Strength Condit Res.

[7] Obesity W. (2023). https://data.worldobesity.org/publications/?cat=19.

[8] Janić M., Janež A., El-Tanani M., Rizzo M. (2025).

[9] Organization WH. WHO European regional obesity report 2022. WHO European Regional Obesity Report 20222022.

[10] Donovan C.M., Buffini M., Walton J., Kehoe L., Kearney J., Flynn A. (2025). Trends in the weight status of adults in Ireland between 1990 and 2024. Eur J Nutr.

[11] Sarma S., Sockalingam S., Dash S. (2021). Obesity as a multisystem disease: trends in obesity rates and obesity‐related complications. Diabetes Obes Metabol.

[12] Lau W.B., Ohashi K., Wang Y., Ogawa H., Murohara T., Ma X.-L. (2017). Role of adipokines in cardiovascular disease. Circ J.

[13] Blüher M. (2019). Obesity: global epidemiology and pathogenesis. Nat Rev Endocrinol.

[14] Obokata M., Reddy Y.N., Pislaru S.V., Melenovsky V., Borlaug B.A. (2017). Evidence supporting the existence of a distinct Obese phenotype of heart failure with preserved ejection fraction. Circulation.

[15] Baker‐Smith C.M., Isaiah A., Melendres M.C., Mahgerefteh J., Lasso‐Pirot A., Mayo S. (2021). Sleep‐disordered breathing and cardiovascular disease in children and adolescents: a scientific statement from the American heart association. J Am Heart Assoc.

[16] Younossi Z., Tacke F., Arrese M., Chander Sharma B., Mostafa I., Bugianesi E. (2019). Global perspectives on nonalcoholic fatty liver disease and nonalcoholic steatohepatitis. Hepatology.

[17] Berenbaum F., Wallace I.J., Lieberman D.E., Felson D.T. (2018). Modern-day environmental factors in the pathogenesis of osteoarthritis. Nat Rev Rheumatol.

[18] Ahmad R., Haque M. (2023). Obesity inflicted reproductive complications and infertility in men. Bangladesh J Med Sci.

[19] Neubronner S.A., Indran I.R., Chan Y.H., Thu A.W.P., Yong E.-L. (2021). Effect of body mass index (BMI) on phenotypic features of polycystic ovary syndrome (PCOS) in Singapore women: a prospective cross-sectional study. BMC Womens Health.

[20] Lauby-Secretan B., Scoccianti C., Loomis D., Grosse Y., Bianchini F., Straif K. (2016). Body fatness and Cancer—viewpoint of the IARC working group. N Engl J Med.

[21] Puhl R., Suh Y. (2015). Health consequences of weight stigma: implications for obesity prevention and treatment. Curr Obes Rep.

[22] Tomiyama A.J., Carr D., Granberg E.M., Major B., Robinson E., Sutin A.R. (2018). How and why weight stigma drives the obesity ‘epidemic’and harms health. BMC Med.

[23] Nunes C.T.L. (2023).

[24] Schauer P.R., Bhatt D.L., Kirwan J.P., Wolski K., Aminian A., Brethauer S.A. (2017). Bariatric surgery versus intensive medical therapy for diabetes—5-year outcomes. N Engl J Med.

[25] Wit M., Trujillo‐Viera J., Strohmeyer A., Klingenspor M., Hankir M., Sumara G. (2022). When fat meets the gut—focus on intestinal lipid handling in metabolic health and disease. EMBO Mol Med.

[26] Valbrun L.P., Zvonarev V. (2020). The opioid system and food intake: use of opiate antagonists in treatment of binge eating disorder and abnormal eating behavior. J Clin Med Res.

[27] Pi-Sunyer X., Astrup A., Fujioka K., Greenway F., Halpern A., Krempf M. (2015). A randomized, controlled trial of 3.0 mg of liraglutide in weight management. N Engl J Med.

[28] Wilding J.P., Batterham R.L., Calanna S., Davies M., Van Gaal L.F., Lingvay I. (2021). Once-weekly semaglutide in adults with overweight or obesity. N Engl J Med.

[29] Lincoff A.M., Brown-Frandsen K., Colhoun H.M., Deanfield J., Emerson S.S., Esbjerg S. (2023). Semaglutide and cardiovascular outcomes in obesity without diabetes. N Engl J Med.

[30] Jastreboff A.M., Aronne L.J., Ahmad N.N., Wharton S., Connery L., Alves B. (2022). Tirzepatide once weekly for the treatment of obesity. N Engl J Med.

[31] Jastreboff A.M., Kaplan L.M., Frías J.P., Wu Q., Du Y., Gurbuz S. (2023). Triple–hormone-receptor agonist retatrutide for obesity—a phase 2 trial. N Engl J Med.

[32] Clément K., van den Akker E., Argente J., Bahm A., Chung W.K., Connors H. (2020). Efficacy and safety of setmelanotide, an MC4R agonist, in individuals with severe obesity due to LEPR or POMC deficiency: single-arm, open-label, multicentre, phase 3 trials. Lancet Diabetes Endocrinol.

[33] Wright D.F., Sinnappah K.A., Hughes D. (2023). Medication adherence research comes of age. Br J Clin Pharmacol.

[34] Craig H.C., Walley D., le Roux C.W. (2024). What influences patient decisions when selecting an obesity treatment?. Obes Pillars.

[35] Fifer S., Keen B., Porter A. (2024). Patient and healthcare professional preferences for prescription weight loss medications in Australia: two discrete choice experiments. Patient Prefer Adherence.

[36] Fischer A.-K., Sadler A., Mathey E., Mühlbacher A. (2025). Analysis of patients preferences in type 2 diabetes mellitus second-line drug treatment: a discrete choice experiment. PLoS One.

[37] Craig H., Alsaeed D., Heneghan H., Al-Najim W., Al Ozairi E., le Roux C. (2024). Factors that determine patients considering medication for the disease of obesity: an IMI2 SOPHIA study. Int J Obes.

[38] Mondoh A., Craig H., Crotty M., Contreras F., le Roux C.W. (2025). How do patients choose between obesity medications: a thematic analysis. Obes Pillars.

[39] Mondoh A., Contreras F., Craig H., Crotty M., le Roux C.W. (2025). Logistics, effectiveness, safety, and accessibility: factors determining obesity medication patient preferences from a photovoice analysis. Obes Pillars.

[40] Flick U. (2022).

[41] Maxwell J.A. (2022).

[42] Rosenstock I. (1974). The health belief model and personal health behavior.

[43] Holt G., Hughes D. (2021). A study using semi‐structured interview and Delphi survey to explore the barriers and enabling factors that influence access and utilisation of weight management services for people living with morbid obesity: a patient and professional perspective. J Hum Nutr Diet.

[44] Wang C., Burris M.A. (1997). Photovoice: concept, methodology, and use for participatory needs assessment. Health Educ Behav.

[45] Omonaiye O., Ananthapavan J., Hensher M., Dinh T.T.N., Pazsa F., Neale S. (2025).

[46] Nyumba T O., Wilson K., Derrick C.J., Mukherjee N. (2018). The use of focus group discussion methodology: insights from two decades of application in conservation. Methods Ecol Evol.

[47] Plummer P. (2017). Focus group methodology. Part 1: design considerations. Int J Ther Rehabil.

[48] Braun V., Clarke V. (2012).

[49] Braun V., Clarke V. (2006). Using thematic analysis in psychology. Qual Res Psychol.

[50] Wharton S., Lau D.C., Vallis M., Sharma A.M., Biertho L., Campbell-Scherer D. (2020). Obesity in adults: a clinical practice guideline. CMAJ (Can Med Assoc J).

[51] Donnan J., Huang R., Twells L. (2022). Patient preferences for attributes of health Canada approved weight loss medications among adults living with obesity in Canada: a qualitative study. Patient Prefer Adherence.

[52] Brown A., Flint S.W., Batterham R.L. (2022). Pervasiveness, impact and implications of weight stigma. eClinicalMedicine.

[53] Ryan L., O'Donoghue G., Crotty M., Birney S., Heary C., Hanlon M. (2024). Factors that influence general practitioners' obesity‐related clinical practices and determinants of behavior to target to promote best practice in obesity care: a qualitative exploration. Obes Sci Pract.

[54] Oshman L., Othman A., Furst W., Heisler M., Kraftson A., Zouani Y. (2023). Primary care providers' perceived barriers to obesity treatment and opportunities for improvement: a mixed methods study. PLoS One.

[55] Lee L. (2025).

[56] Gala K., Gawey B., Hargraves I., Brito J.P., Acosta A. (2023). S1656 development of a shared decision-making tool for obesity management. Off J Am Coll Gastroenterol ACG.

[57] Kim D.D., Hwang J.H., Fendrick A.M. (2024). Balancing innovation and affordability in anti-obesity medications: the role of an alternative weight-maintenance program. Health Affairs Scholar.

